# Impedance and Dielectric Properties of PVC:NH_4_I Solid Polymer Electrolytes (SPEs): Steps toward the Fabrication of SPEs with High Resistivity

**DOI:** 10.3390/ma15062143

**Published:** 2022-03-15

**Authors:** Muaffaq M. Nofal, Shujahadeen B. Aziz, Hewa O. Ghareeb, Jihad M. Hadi, Elham M. A. Dannoun, Sameerah I. Al-Saeedi

**Affiliations:** 1 Department of Mathematics and Science, Prince Sultan University, P.O. Box 66833, Riyadh 11586, Saudi Arabia; muaffaqnofal69@gmail.com; 2 Hameed Majid Advanced Polymeric Materials Research Lab., Physics Department, College of Science, University of Sulaimani, Qlyasan Street, Sulaimani 46001, Kurdistan Regional Government, Iraq; 3 Department of Civil Engineering, College of Engineering, Komar University of Science and Technology, Sulaimani 46001, Kurdistan Regional Government, Iraq; 4 Department of Chemistry, College of Science, University of Sulaimani, Qlyasan Street, Sulaimani 46001, Kurdistan Regional Government, Iraq; hewa.ghareeb@univsul.edu.iq; 5 Department of Medical Laboratory of Science, College of Health Sciences, University of Human Development, Sulaimani 46001, Kurdistan Regional Government, Iraq; jihad.chemist@gmail.com; 6 Department of Mathematics and Science, Woman Campus, Prince Sultan University, P.O. Box 66833, Riyadh 11586, Saudi Arabia; elhamdannoun1977@gmail.com; 7 Department of Chemistry, College of Science, Princess Nourah bint Abdulrahman University, P.O.Box 84428, Riyadh 11671, Saudi Arabia; sialsaeedi@pnu.edu.sa

**Keywords:** PVC polymer, NH4I salt, solid polymer electrolytes, low dielectric constant, impedance study

## Abstract

In the present article, a simple technique is provided for the fabrication of a polymer electrolyte system composed of polyvinyl chloride (PVC) and doped with varying content of ammonium iodide (NH_4_I) salt using solution-casting methodology. The influences of NH_4_I on the structural, electrochemical, and electrical properties of PVC have been investigated using X-ray diffraction, electrochemical impedance spectroscopy (EIS), and dielectric properties. The X-ray study reveals the amorphous nature of the polymer–salt complex. The EIS measurement revealed an ionic conductivity of 5.57 × 10^−10^ S/cm for the electrolyte containing 10 wt.% of salt. Our hypothesis is provided, which demonstrated the likelihood of designing highly resistive solid electrolytes using the concept of a polymer electrolyte. Here, the results showed that the resistivity of the studied samples is not dramatically decreased with increasing NH_4_I. Bode plots distinguish the decrease in resistance or impedance with increasing salt contents. Dielectric measurements revealed a decrease in the dielectric constant with the increase of NH_4_I content in the PVC polymer. The relaxation time and dielectric properties of the electrolytes confirmed their non-Debye type behavior. This pattern has been validated by the existence of an incomplete semicircle in the Argand plot. Insulation materials with low *ε*_r_ have found widespread applications in electronic devices due to the reduction in delay, power dissipation, and crosstalk. In addition, an investigation of real and imaginary parts of electric modulus leads to the minimized electrode polarization being reached.

## 1. Introduction

Dielectric materials with outstanding properties, such as high *ε*′, low *ε*″, and high electric breakdown strength are desired eagerly for their potential energy storage application [1]. The research on polymer materials has further enhanced with the advances in science and technology. However, the difficulties in synthesis strategies, precursor sources, and production costs are encountered with novel-polymer-type development [2]. The data of the dielectric properties provide information on the chemical and physical characteristics of polymers. Moreover, these characteristics can be significantly affected by a polymer- or dopant-blending procedure [3]. Two main methods are available to achieve polymeric materials with low dielectric constant (low-k). The first method involves the reduction of molecular polarizability by opting for the suitable chemical elements for preparing such materials. Those atoms with appropriate electronegativity are exploited to induce molecular polarizability, and two factors have been taken into consideration. Firstly, the incorporation of the optimum number of C–C constitutional units that possess relatively low polarizability in the polymer backbone produces low-k. Secondly, the polarization process is highly dependent on the nature of molecular polarizability. It is worth mentioning that a low-density dielectric material configuration correlates to a low-density component [4]. Previous studies demonstrated that salt added into polymers such as chitosan, methylcellulose, dextran, polyethylene oxide, and polyvinyl alcohol are crucial to reducing the resistivity of polar polymers and increasing the DC conductivity owing to salt dissociation by the host polymer functional groups [5,6,7,8,9,10,11,12,13,14]. Herein, the material of use is PVC host polymer that experiences several different behaviors, making it remarkably different from that previously reported for polymer electrolyte (PEs) materials.

In order to develop a new class of materials in microelectronic applications, a variety of low-k polymer materials have been prepared. One of the well-established criteria for evaluating polymer materials is having low-k (*ε*′ < 2) for use in microelectronic circuits. Dielectric constants with values less than 2.5 are specified to have “no known solution” [15]. Previously, the use of electrical impedance spectroscopy has been confirmed as a unique tool suitable for studying the electrical and dielectric properties of polymeric materials [16,17,18,19,20]. An innovative strategy to create low-k polymeric film is presented in this paper. On this matter, we discovered that our polymer-based samples exhibit low-k of *ε*′ = 1.15 at high frequencies, which can be considered as a novel way for designing polymers with low dielectric constants. As far as we know, this is the first article that demonstrates the limitations of a PVC polymer in the preparation of PEs, but it may also be considered as a first report on the usage of PEs with high resistance and low-k.

## 2. Methodologies

### 2.1. Polymer Bend Preparation

**Scheme 1:**
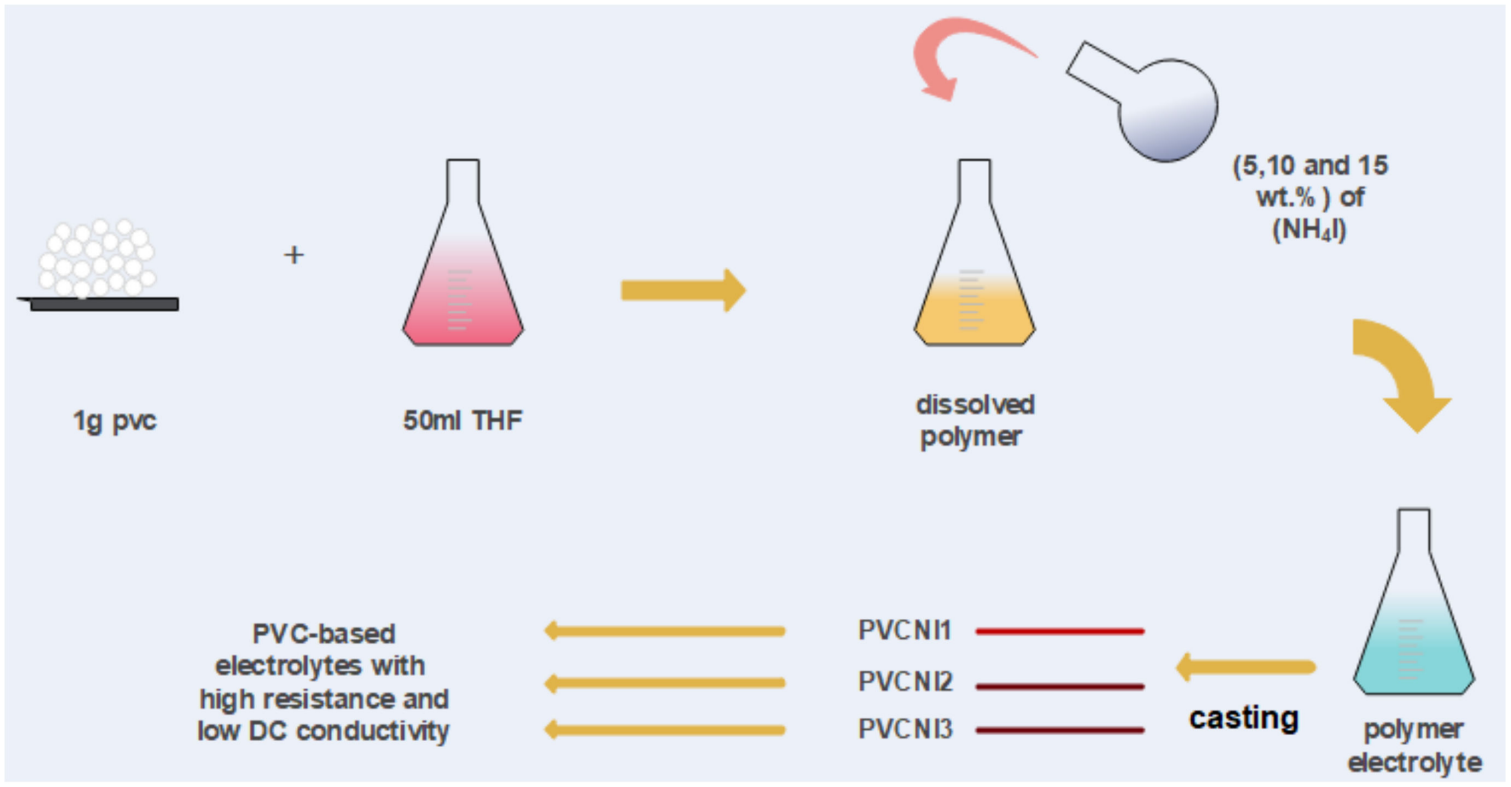
Experimental procedure for PVC based electrolyte preparation.

In this study, polyvinyl chloride (PVC) was obtained from Sigma-Aldrich and used as a starting material for making the polymer films by utilizing the solution-cast technique. Thus, 1 g of PVC was dissolved in 50 mL of tetra hydro furan (THF). Afterward, NH_4_I salt was added to the PVC solution at varying amounts of 0, 5, 10, and 15 wt.% and the resulting samples were coded as PVCNI0, PVCNI1, PVCNI2, and PVCNI3, respectively. A number of dry plastic Petri dishes were used to make films from the mixtures by pouring and then leaving them to dry at room temperature (RT) to reach solvent-free films. Scheme 1 shows experimental methodology. Additionally, the films were maintained in desiccators containing a blue silica gel desiccant.

### 2.2. Characterization Techniques

The amorphousness and crystallinity nature of the prepared SPE films were determined using the X-ray diffraction technique. At room temperature, a Siemens D5000 X-ray diffractometer with a working potential of 40 kV and operating current of 40 mA was used. Moreover, the electrolyte films were evaluated via monochromic X-rays with a wavelength of 1.5406 Å with two angles (2θ) 10° to 80° with a step size of 0.1. An HIOKI 3531 Z LCR Hi-tester was used to measure the films’ electrical impedance in the frequency range of 50 Hz to 5000 kHz at ambient temperature. The LCR meter was connected to a data-collecting software on a computer, which allowed the real and imaginary parts of the impedance to be determined. Under spring pressure, discs of 2 cm diameter prepared from the polymer blend films were put in between two identical circular stainless steel electrodes to ascertain a good contact.

## 3. Results and Discussion

### 3.1. X-ray Difraction Study

**Figure 1:**
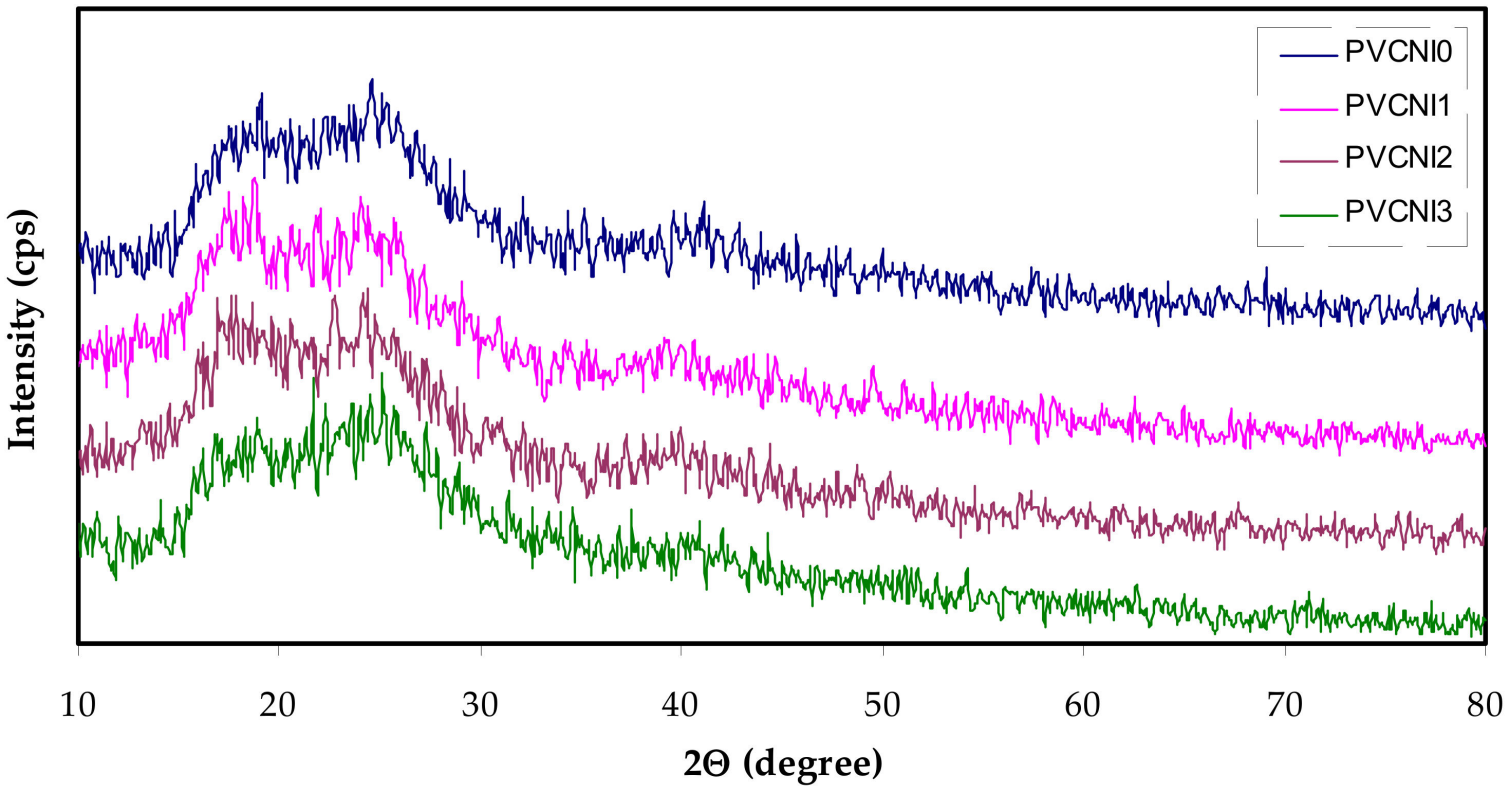
X-ray pattern neat PVC (PVCNI0) and PVC-based electrolytes (PVCNI1, PVCNI2 and PVCNI3).

The structural behavior of solid polymer electrolytes based on PVC-NH4I has been carried out using an X-ray diffraction pattern. Figure 1 demonstrates the XRD pattern for the neat PVC and PVC doped with various amounts of NH4I salt. Previous studies established that XRD is crucial to examine the complexation of salt or fillers with polymer host materials [21,22]. The presence of broad peaks in the PVC: NH_4_I system indicates the amorphous behavior. The appearance of many crystalline peaks was documented by Jyothi et al. [23] for PVC-based electrolytes. The amorphous behavior of PVC suggests that adding NH_4_I salt reduces the crystalline phase [24]. Overall the broadness of PVC: NH_4_I is higher than that of neat PVC. The absence of sharp crystalline peaks in PVC: NH_4_I is evidence for the fact that NH_4_I is complexed with PVC structure. In later sections, we will show our hypothesis regarding the PVC: NH_4_I complexation.

### 3.2. Impedance Study

**Figure 2:**
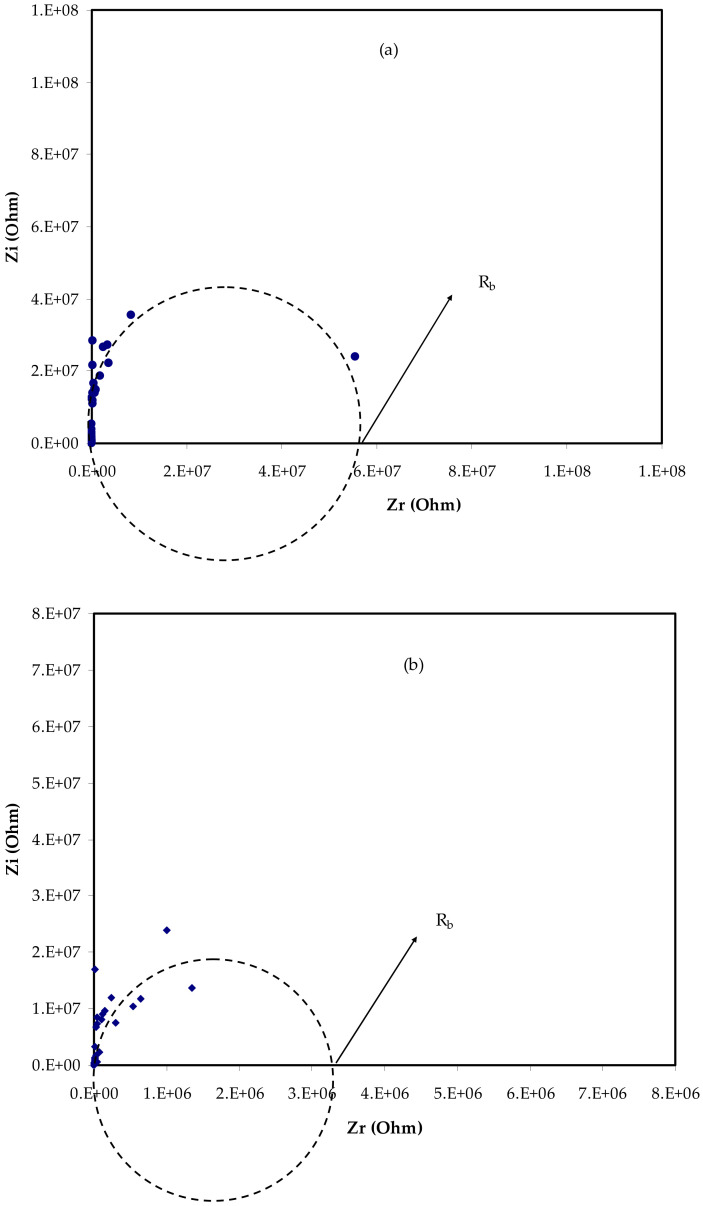

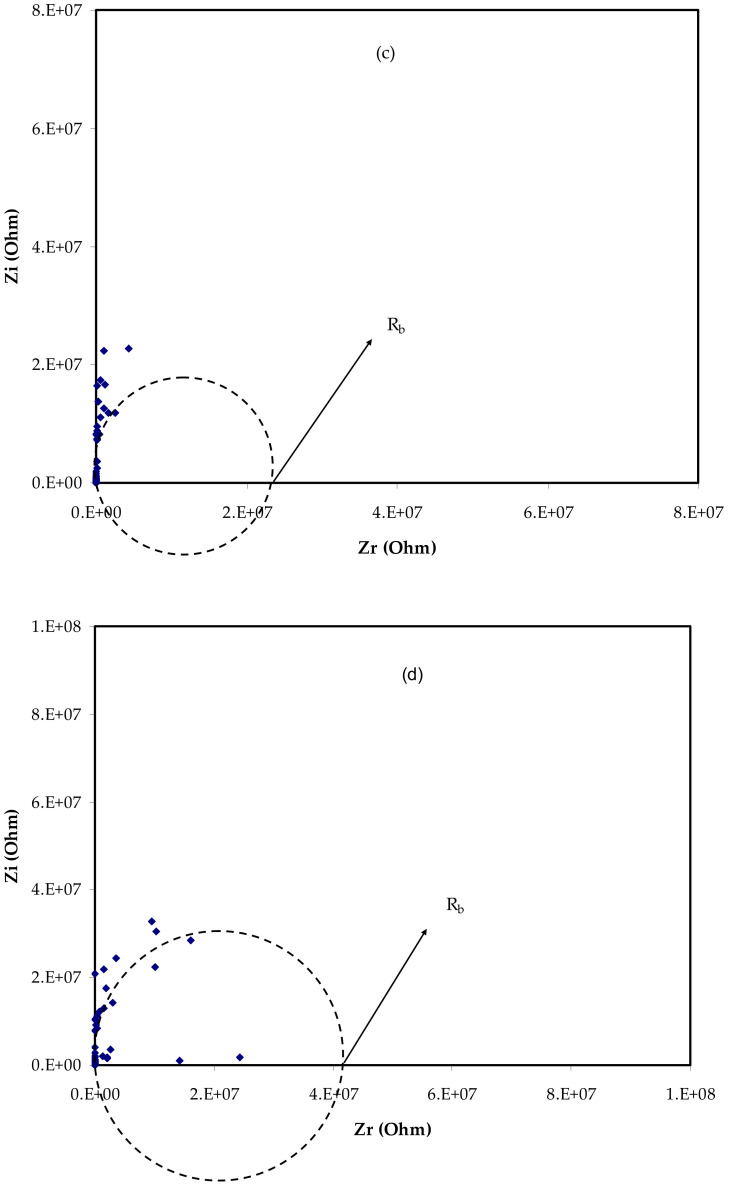
EIS plots for (**a**) PVCNI0, (**b**) PVCNI1, (**c**) PVCNI2, and (**d**) PVCNI3 electrolytes.

Figure 2a–d depicts the electrical impedance plots for the prepared electrolyte films at ambient temperature, i.e., complex impedance’s real (Z_r_) and imaginary (Z_i_) parts. Electrochemical impedance spectroscopy (EIS) is used to examine the polymeric chains, mobility, and carrier formation mechanisms to establish the conduction mechanism. The conductivities of polymer complexes can be calculated using Equation (1) and the bulk resistance (*R*_b_) obtained from the impedance plots’ intercept with the real axis, as well as electrolyte dimensions [25].

(1) $$\sigma_{\mathrm{dc}}=\left(\frac{1}{R_{b}}\right)\times \left(\frac{t}{A}\right)$$

**Figure 3:**
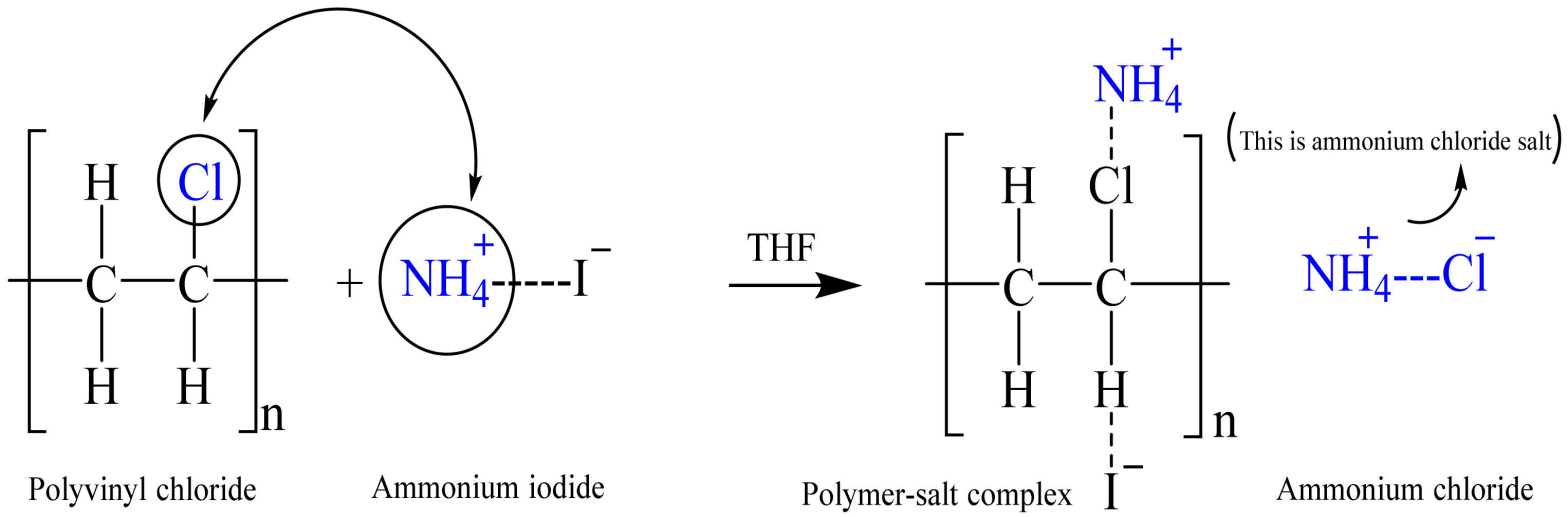
Schematic illustration of interaction between PVC host polymer and NH_4_I salt. The tight binding of NH_4_ cation with Cl-side chain of PVC backbone is responsible for low conductivity of PVC-based electrolyte.

Here, *t* is the film thickness, and *A* is the area of the electrodes. Table 1 lists the DC conductivity of the films. As is obvious from this table, the excess salt does not lead to an increase in the DC conductivity. In this piece, there is no apparent semicircle, and all of the films route the data to the Y-axis. As a result, the system seems to act in a similar way to a high-resistance capacitor. As a consequence, at room temperature, ionic conductivity is diminished. A polymer electrolyte based on a PVC host has poor conductivity, according to prior studies. The primary cause for this is that the functional group (i.e., chlorine atom) is attached to the chain. The poor ionic conductivity seen in Figure 3 illustrates our hypothesis. NH_4_Cl salt is formed when chlorine atoms interact with salt’s cations (NH_4_^+^), creating ammonium chloride. Ionic conductivity is reduced because the cation has a difficult time transitioning between functional groups in this situation. The tight binding of NH_4_ cation with the Cl-side chain of PVC backbone restricts the cation motion which is responsible for DC conductivity. This results in a considerable reduction in free-ion concentration and significantly slows ion transport between adjacent sites of the host polymer, lowering the electrolyte’s ionic conductivity. As a consequence, NH_4_I cannot be used to increase conductivity, preventing an impedance plot spike near enough to the EIS plot’s imaginary region. To our knowledge and based on our hypothesis shown in Figure 3, polymers having F, Br, or Cl side chains are useful to fabricate high resistance polymer electrolytes due to the formation of salt inside the host polymer. The maximum ionic conductivity of ~10^−6^ S/cm was found in our earlier work for the glycerol-plasticized system of (PVC) doped with ammonium thiocyanate (NH_4_SCN), which it is higher compared to this outcome. This could be attributed to the action of glycerol as a plasticizer, which offers more channels for ions to easily pass through the polymer matrix [26]. Additionally, Subban et al. [27] have stated the ionic conductivity of ~10^−6^ S/cm for their electrolyte system based on PVC-hexanoyl chitosan blend system doped with lithium triflate.

### 3.3. Bode Plot Analysis

**Figure 4:**
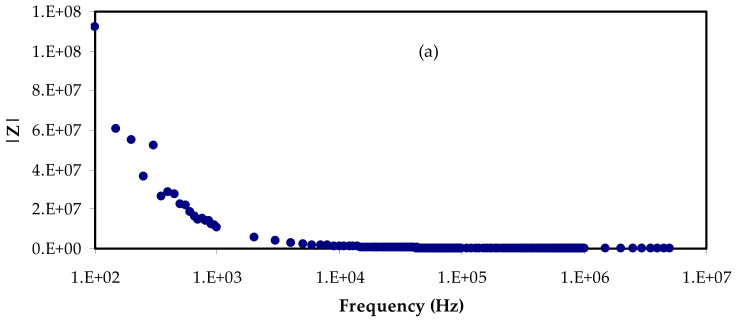

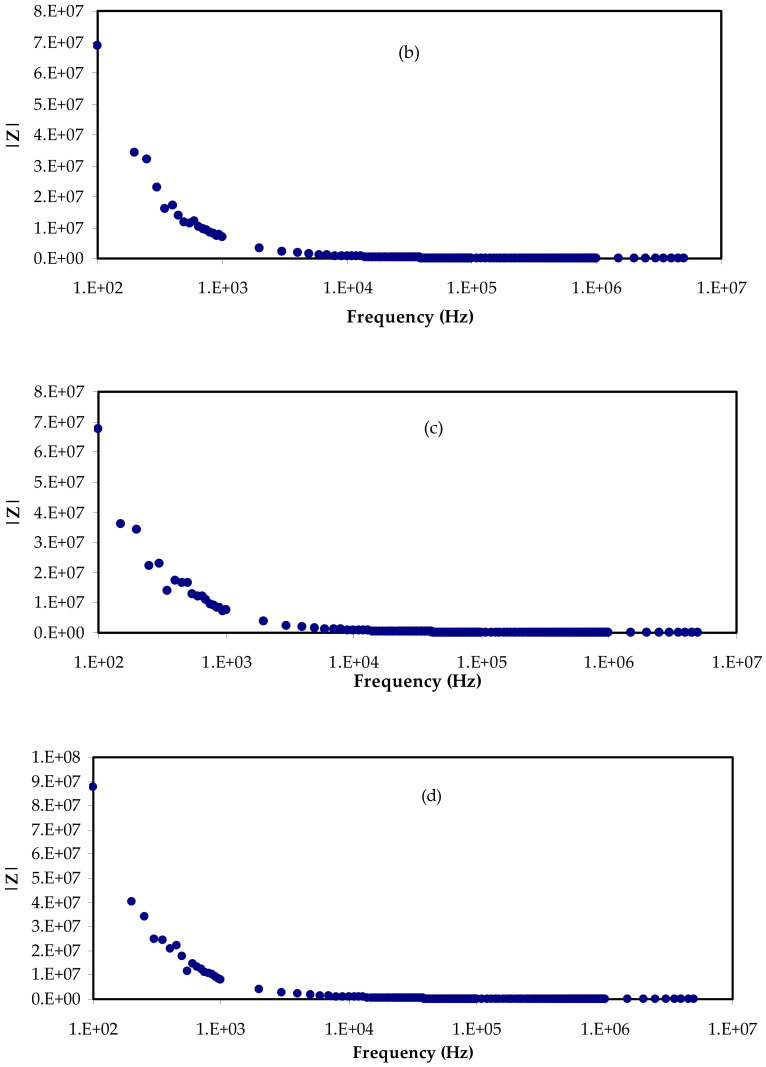
Bode plots for (**a**) PVCNI0, (**b**) PVCNI1, (**c**) PVCNI2, and (**d**) PVCNI3 electrolytes.

Studying the Bode graphs also confirms the previous explanation and suggested Nyquist semicircles. Figure 4 illustrates the Bode graphs for the created PVC: NH_4_I systems at room temperature. The Bode graphs should have three separate zones, according to earlier research: capacitive, diffusion, and charge-transfer [28]. At very low frequencies, such as 10^−2^ to 100 Hz, the capacitive or plateau area can be seen. As a consequence, measurement frequency limitations account for the absence of this part in the Bode graph. Ion transfer in the amorphous phase of electrolytes is represented by the semicircle in the impedance plots (see Figure 2), whereas Warburg’s contribution is represented by the tails. The charge transfer resistance decreased as the amount of NH_4_I salt was increased, as seen in Figure 4. The low-frequency dispersion region in the Bode plot is ascribed to ion diffusion, while the high-frequency region is related to charge transport resistance. These results indicate that the produced polymer electrolytes have high resistance. The electrolyte is the foundation of energy storage and conversion devices, such as batteries, supercapacitors, solar cells, fuel cells, etc. Producing polymer electrolytes with a high DC ionic conductivity is essential from a physics standpoint. From the chemist’s viewpoint, it is important that the films have a low charge-transfer resistance. Therefore, the produced electrolyte is not appropriate for energy-related applications because of its high-resistive behavior. However, this so-called highly resistive electrolyte can be used to make electrolytic resistors, which have a wide range of uses in the sensor sector.

### 3.4. Dielectric Properties

The studies of dielectric relaxation are helpfully survey in understanding the performance of polymers. To examine dipole relaxation in polymeric materials, dielectric relaxation spectroscopy was accomplished over a large frequency range [29]. Using the following equations, the real and imaginary parts of ε* and M* may be calculated from the real (*Z*′) and imaginary (*Z*″) parts of Z* [9,30,31]:

(2) $$\varepsilon_{r}=\frac{Z_{i}}{\omega C_{o}(Z_{r}{}^{2}+Z_{i}{}^{2})}$$

(3) $$\varepsilon_{i}=\frac{Z_{r}}{\omega C_{o}(Z_{r}{}^{2}+Z_{i}{}^{2})}$$

(4) $$M^{\prime }\;=\frac{\varepsilon^{\prime }}{\left({\varepsilon^{\prime }}^{2}+{\varepsilon^{\prime \prime }}^{2}\right)}=\omega C_{o}Z_{i}$$

(5) $$M^{\prime \prime }\;=\frac{\varepsilon^{\prime \prime }}{\left({\varepsilon^{\prime }}^{2}+{\varepsilon^{\prime \prime }}^{2}\right)}=\omega C_{o}Z_{r}$$

**Figure 5:**
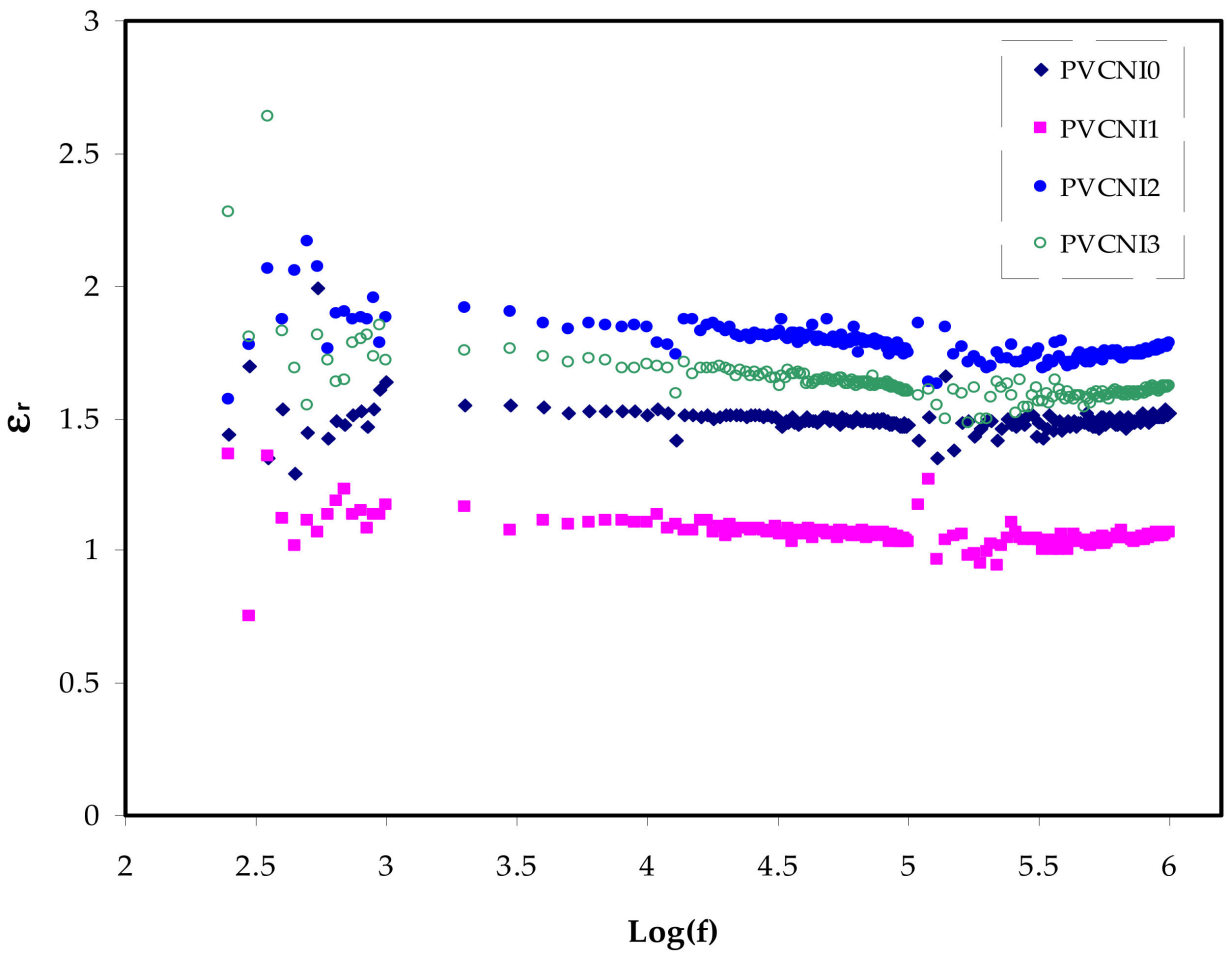
*ε*_r_ versus log (*f*) for all the electrolyte samples.

**Figure 6:**
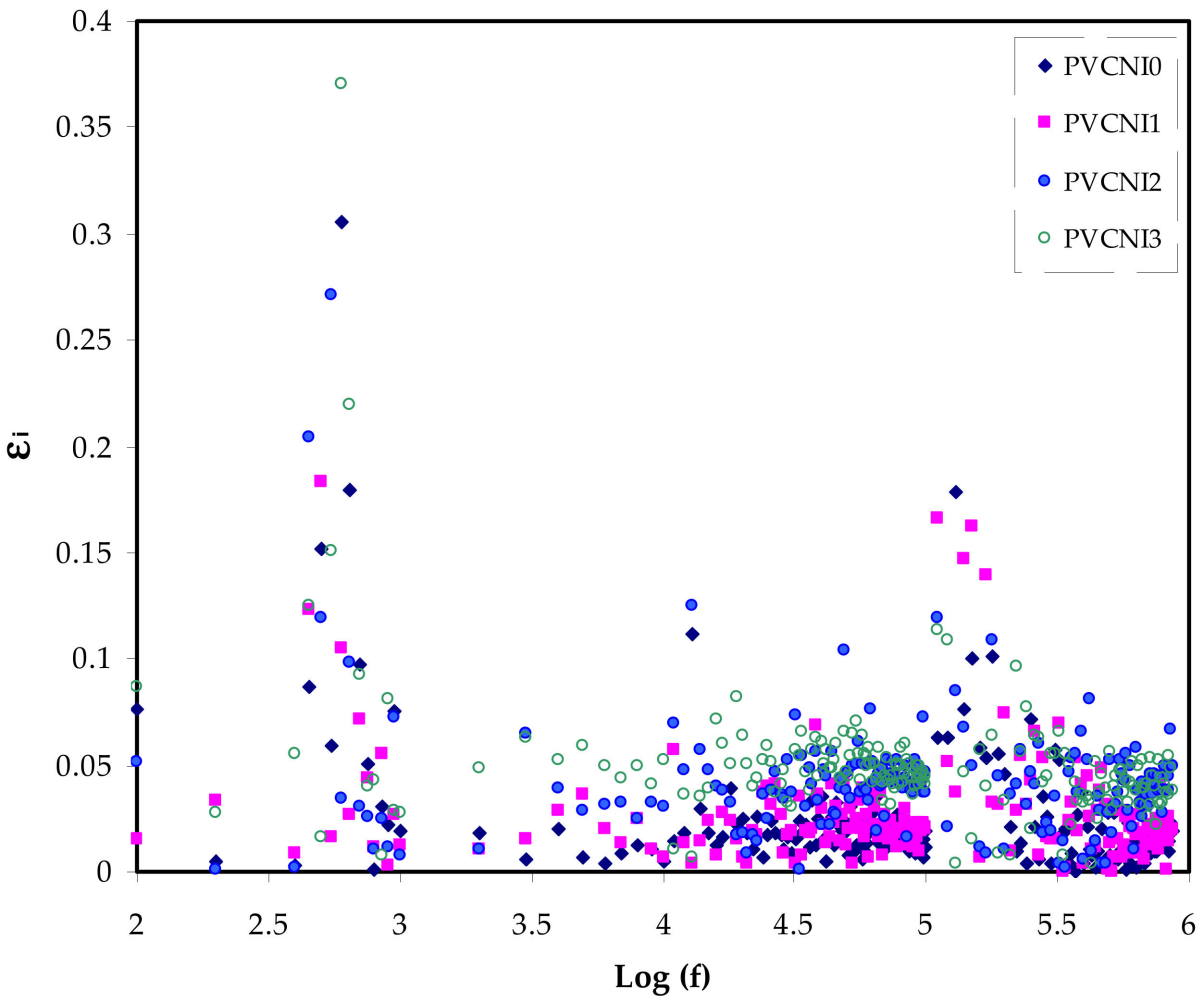
*ε*_i_ versus log (*f*) for all the electrolyte samples.

Here, *C_o_* is the vacuum capacitance of the cell used in the measurements, which equals ε_o_A/t (where *t* and *A* are the thickness and area of the film, respectively), and *ω* = 2π*f* is the angular frequency, *f* is the frequency in hertz. Figure 5 and Figure 6 show the spectra of dielectric constant and dielectric loss, both against frequency for all the samples. Figure 5 demonstrates the dielectric constant of the PVC system recording relatively low as NH_4_I salt is added. Through the entire range of frequency, the *ε*′ is independent on frequency change. The ε′ value of the electrolytes at the high-frequency region is estimated using the intersection of the lines. A recent article has introduced two parameters in describing the dielectric constant of a polymer [32], namely the molecular polarizability, which is induced by varying polarizable groups and the free volume associated with the polymer. Speed up of transistors and the necessity for miniaturization in order to pack a large number of transistors onto a microchip has contributed to the advancements in microelectronic integrated systems during the previous few decades. In today’s electronics industry, low-k insulating materials are critical [33]. Scientists can minimize crosstalk noise, signal delays, and power consumption in integrated circuits by using high-performance polymers with low-k and loss factors [34]. The Semiconductor Industry Association (SIA) roadmap states that the smallest feature size has ever been made is about 0.15 µm by 2001. To achieve this size, it is required to have a dielectric material with k = 2.3 for insulation. Nowadays, an ultra-low dielectric material (k < 2) has to be prepared for the next generation of an integrated circuit [35]. Low DC and *ε*′ values have been attributed to other factors, such as crystalline phases [36,37]. The combination of NH_4_^+^ and Cl^−^ of the PVC chain is more likely in the current study than the abovementioned factors.

**Figure 7:**
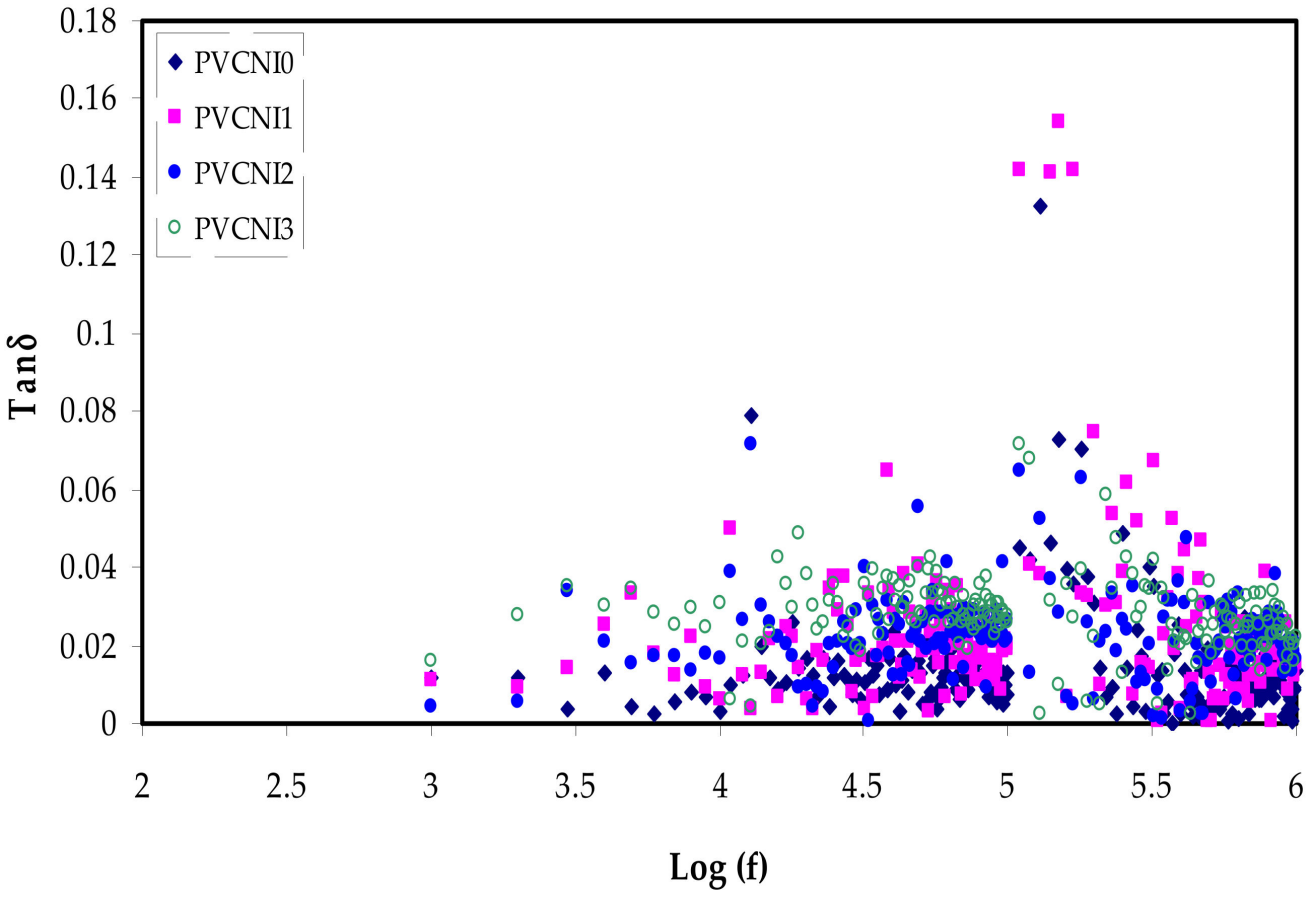
Tanδ versus log (*f*) for all the electrolyte samples.

The tangent loss was carried out to further understand the process of ionic mobility and molecular interaction for the different amounts of NH_4_I salt with the PVC systems [38]. Figure 7 depicts the loss tangent spectra for these systems as a function of frequency at ambient temperature. The occurrence of a dielectric relaxation process was demonstrated by the tangent loss peaks and their shift at the various weight ratios of NH_4_I salt [39]. It is noteworthy that, at a higher frequency, the tangent loss is increased and reaches a peak, followed by a decline when the frequency has increased further. The frequency associated with the highest tangent loss value was discovered to move to the high frequency. The data shifts to high frequency suggest a dielectric relaxation process [40]. This is also comparable with the theoretically expected outcome based on the Debye equation [41].

### 3.5. Electric Modulus Study

**Figure 8:**
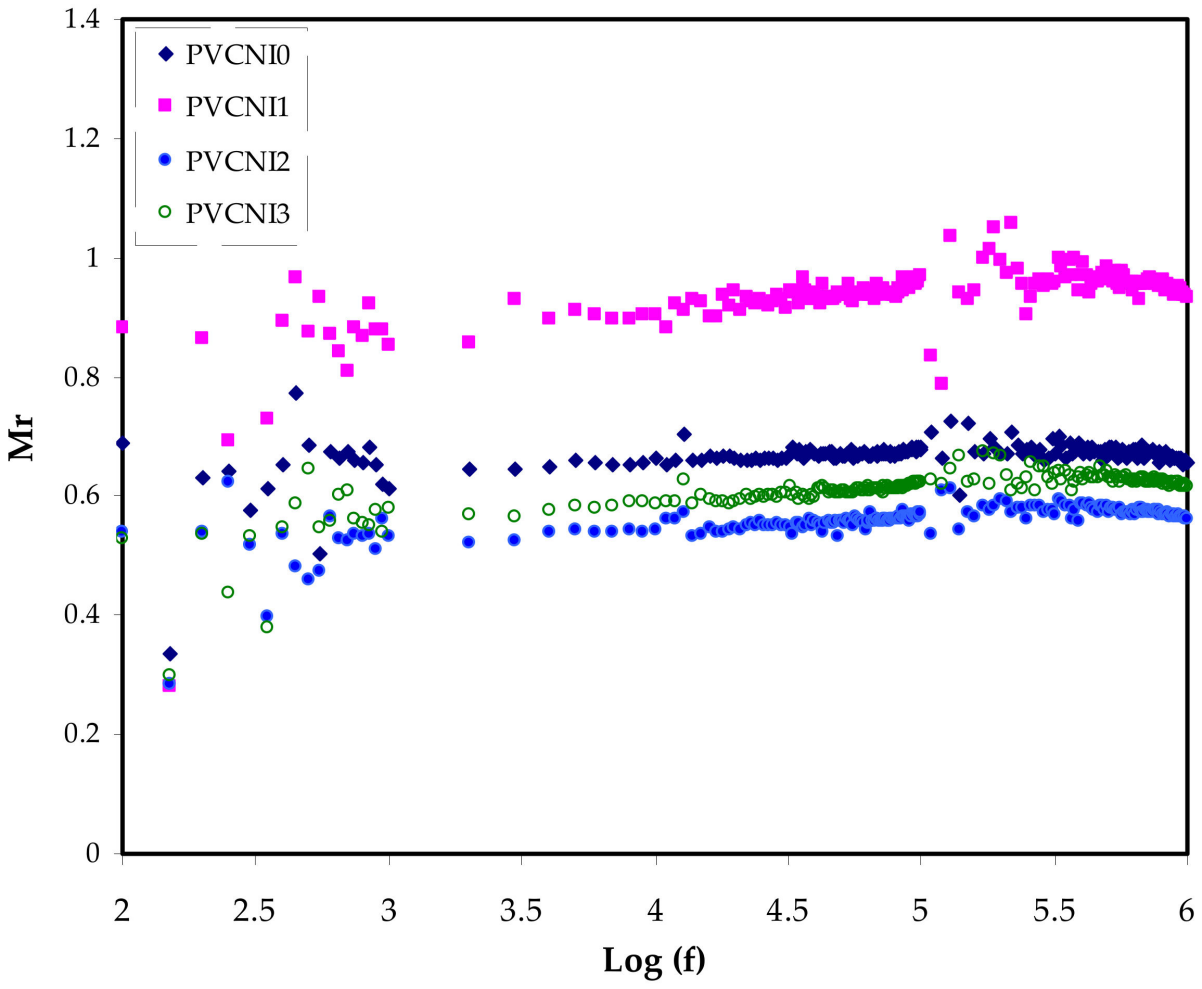
*M*_r_ versus log (*f*) for all the electrolyte samples.

**Figure 9:**
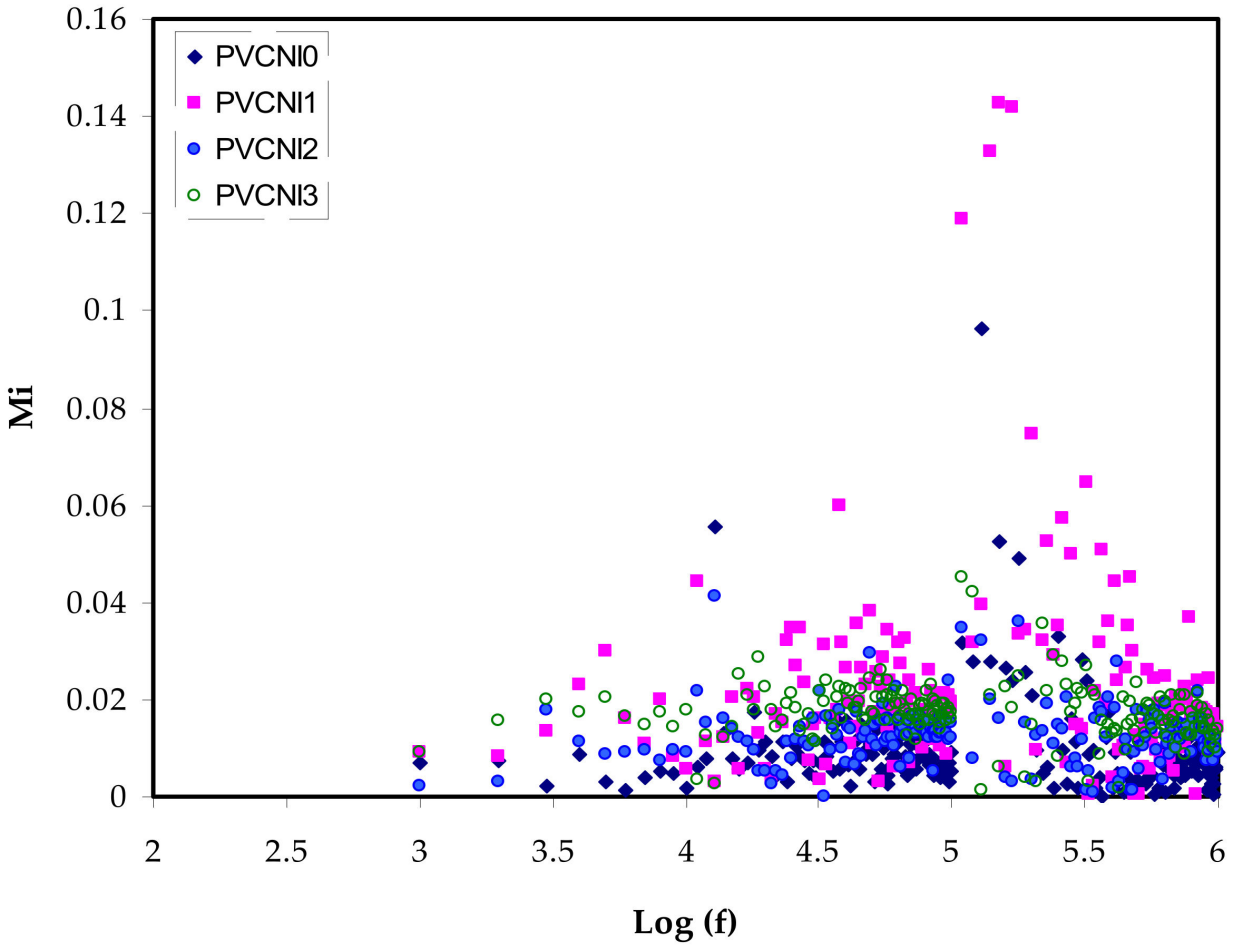
*M*_i_ versus log (*f*) for all the electrolyte samples.

Presently, the M* illustration is commonly employed to probe ionic conductivities concerning ionic process-conductivity relaxation time relations [42]. To enable a realization of the relaxation, it is worth selecting the electric modulus formalism over the permittivity. Furthermore, the conductivity-relaxation approaches prevent substantial variations in the values of *ε*′ and *ε*″ at low frequencies. The dielectric spectrum analysis concern may be solved by neglecting the injection of space charges and the absorption of impurities [43]. At low frequencies, the enormous capacitance of the double-layer charges causes the *M*′ and *M*″ values to go to zero, suggesting the removal of polarization, as seen in Figure 8 and Figure 9 [44,45]. When compared to the dielectric constant pattern, the *M*′ spectra follow an entirely different path. Figure 5 shows a high dielectric-constant value at low frequencies. The complex dielectric constant (Cdc) is shown to have a minimum value at high frequencies in respect to the *M*′ and *M*″ modules. The imaginary portion of modulus spectra is seen in Figure 9. The relaxation peaks of conductivity may be seen here. As the NH_4_I salt level rises, the data have shifted toward lower frequency areas. This indicates that the system behaves almost as a capacitor with high resistance. Segmental mobility decreases with an increase in relaxation time in the amorphous phase of the samples. In the shift from long-range translational ionic motion (translational mobility) to short-range segmental motion (dipolar mobility), the carriers are restricted to potential wells and traverse only a limited distance. These peaks show the change [44,46]. The location of the rearrangement of dipole within molecules corresponds to the alternating electric field is the place where polarization occurs. This response can easily be recognized on the low-frequency side of the peaks. Correspondingly, a dual capacitance layer will be formed between the electrode and the electrolyte film. The consequence of this phenomenon is an emergence of a relatively high dielectric constant and in an extremely tiny *M*″. The high-frequency side of the peak is commonly specified for a stimulus of the ions that can only move locally (reorientation) [47]. The presence of a peak in *M*″ and absence in *ε*″ spectra, respectively (see Figure 6) implies the large extent of a bounded ion with the polymer segmental motion [48,49]. In the *M*″ plot, the peaks are characterized by asymmetric shapes on both sides of the maxima, verifying the nonideal Debye behavior.

**Figure 10:**
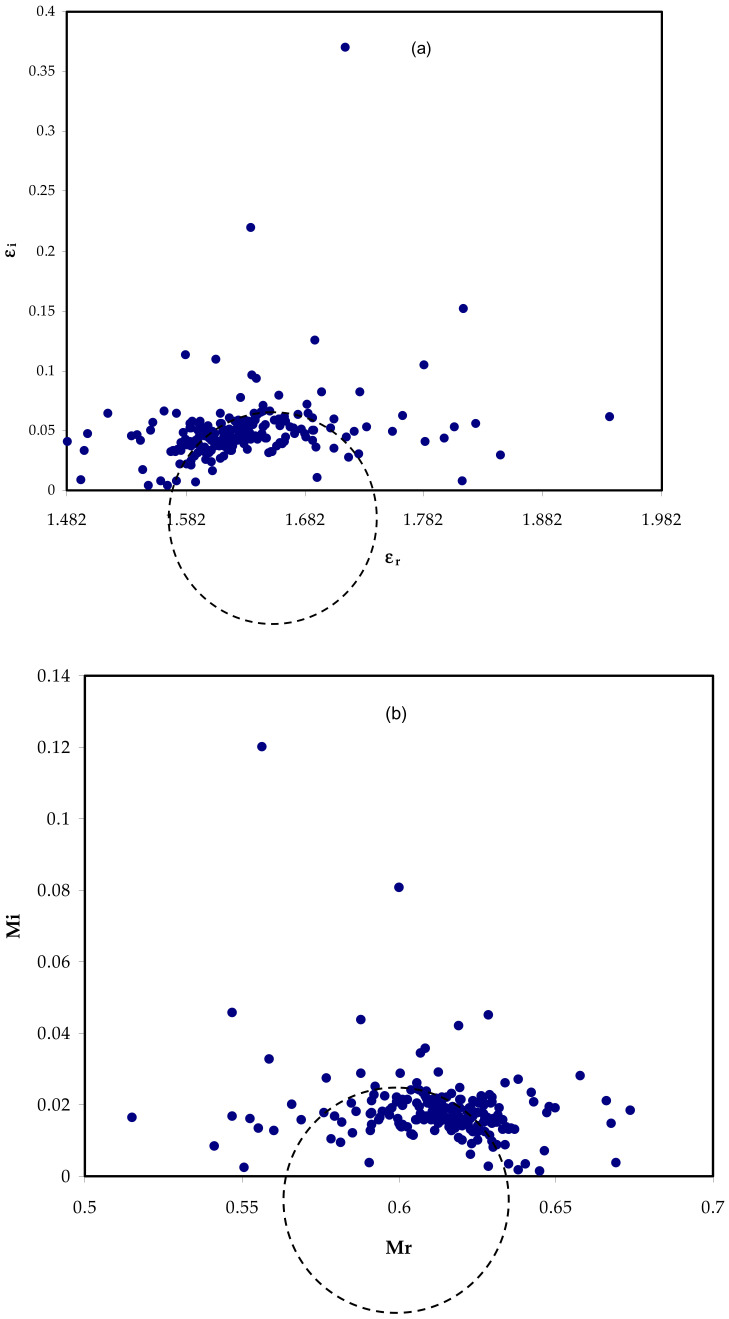
Argand plots (**a**) *ε*_r_ versus *ε*_i_ and (**b**) *M*_r_ versus *M*_i_.

The relaxation behavior in the present polymer electrolyte can be demonstrated by examining the Argand plot. Figure 10 shows one incomplete semicircle (IS) that was recorded for the Argand plots for PVCNI3 system. From *ε*_i_ vs. *ε*_r_ (Figure 10a) and *M_i_* vs. *M_r_* (Figure 10b), one can notice that the tail cannot be observed in the Argand plots which reveals the resistive behavior of the electrolyte. Earlier reports revealed a long tail in Argand plots for ion-conducting polymer electrolytes [5,28]. The incomplete semicircle in the Argand plots (Figure 10) indicated a non-Debye-type relaxation process. As a general principle, the Debye model is established for noninteracting identical dipoles. Thus, the non-Debye behavior originates from real materials that experience several types of polarization mechanisms, which in turn cause several numbers of interactions between ions and dipoles. The Argand plot with a full semicircular arc is widely accepted to be associated with ion relaxation. This would largely be attributed to the conductivity relaxation process and partly due to the viscoelastic relaxation process within polymer chain motion promoting ion translation [49,50,51].

## 4. Conclusions

In conclusion, polymers with Cl, F or Br side chains are useful to fabricate polymer electrolytes with high resistivity and low *ε*_r_-value, utilizing a solution-casting procedure. The results of impedance and dielectric properties reveal the importance of this method to produce polymer/salt films for insulation applications, especially in microelectronics due to their low dielectric constant (*ε*_r_-value < 2) and high charge transfer resistance. The structural and electrical properties were investigated using different tools. The amorphous nature of the polymer–salt complex is revealed by the XRD analysis. The experimental electrical impedance spectra for the samples provide deep knowledge about the charge-transfer resistance. The EIS study illustrates the fact that the resistivity of the samples did not drastically drop with an increase in the quantity of NH_4_I. It was discovered that increasing the concentrations of NH_4_I did not decrease charge-transfer resistance. The EIS measurement revealed an ionic conductivity of 5.57 × 10^−10^ S/cm for the electrolyte containing 10 wt.% of salt, which is close to the range of insulators. The tight association of cations with the polymer’s side chains forms a salt inside the polymer structures. The conductivity trends were validated by the dielectric study. The dielectric constant study of the PVC polymer is lowered after incorporation of relatively large amount of NH_4_I salt. From the electric modulus study, it is proved that the electrode polarization was minimized. The relaxation behavior was examined by the Argand plot. The IS arc in the Argand plots demonstrates a non-Debye-type relaxation process for ions.

## Figures and Tables

**Table 1:** DC conductivity of the systems at RT.

Symbols	R_b_ (Ohm)	DC Conductivity (S/cm)
PVCNI0	61,000,000	2.28 × 10^−10^
PVCNI1	32,100,000	4.33 × 10^−10^
PVCNI2	25,000,000	5.57 × 10^−10^
PVCNI3	42,500,000	3.27 × 10^−10^

## Data Availability

The data presented in this study are available on request from the corresponding author.
